# Interaction effects between sleep-related disorders and depression on hypertension among adults: a cross-sectional study

**DOI:** 10.1186/s12888-024-05931-9

**Published:** 2024-07-02

**Authors:** Chunhua Liu, Zegen Ye, Liping Chen, Huaqiang Wang, Binbin Wu, Di Li, Sisi Pan, Weiwen Qiu, Haiqin Ye

**Affiliations:** 1 Department of Rehabilitation, Lishui Hospital of Traditional Chinese Medicine, Affiliated to Zhejiang University of Chinese Medicine, No. 800 Zhongshan Street, Liandu District, Lishui City, Zhejiang Province China; 2 Lishui Central Hospital, No. 289 Kuocang Road, Liandu District, Lishui City, Zhejiang Province China; 3 Department of Neurology, Lishui Hospital of Traditional Chinese Medicine, Zhejiang University of Chinese Medicine, 800 Zhongshan Street, Lishui City, Zhejiang 323000 China; 4 Department of Clinical Training, Lishui Municipal Central Hospital, Lishui, Zhejiang Province 323000 China

**Keywords:** Hypertension, Sleep disorders, Depression, Interaction

## Abstract

**Background:**

Hypertension, sleep disorders, and depression represent notable public health issues, and their interconnected nature has long been acknowledged. The objective of this study is to explore the interplay between sleep disorders and depression in the context of hypertension.

**Methods:**

This cross-sectional study involved 42,143 participants aged 18 and above from the NHANES database across seven survey cycles between 2005 and 2018. After excluding those with missing data on depression, sleep disorders, and hypertension, as well as incomplete main variables, 33,383 participants remained. We used weighted logistic regression to examine the relationship between sleep disorders, depression, and hypertension. Additionally, we assessed the interaction between sleep disorders and depression on hypertension using both multiplicative and additive approaches to quantify their combined effect.

**Results:**

Compared to individuals without sleep disorders, those with sleep disorders have an increased risk of hypertension (OR = 1.51, 95% CI: 1.37–1.67). Furthermore, individuals with depression experience a significantly higher risk of hypertension compared to those with sleep disorders alone (OR = 2.34, 95% CI: 1.95–2.80). Our study reveals a positive interaction between sleep disorders and depression in relation to hypertension risk (OR = 1.07, 95% CI: 1.02–1.13). In addition, we observed the quantitative additive interaction indicators (RERI = 0.73, 95% CI: 0.56 ~ 0.92; API = 0.31, 95% CI: 0.11 ~ 0.46; SI = 2.19, 95% CI: 1.08–3.46) influencing hypertension risk. Furthermore, our research also identified that individuals with less than 7 h of sleep, a sleep latency period between 5 and 30 min, or a latency period exceeding 30 min experience a significantly increased risk of hypertension.

**Conclusions:**

Our research uncovered separate links between sleep disorders, depression, and hypertension prevalence. Moreover, we identified an interaction between depression and sleep disorders in hypertension prevalence. Enhancing mental well-being and tackling sleep disorders could help prevent and manage hypertension. Yet, more investigation is required to establish causation and clarify mechanisms.

**Supplementary Information:**

The online version contains supplementary material available at 10.1186/s12888-024-05931-9.

## Background

Hypertension, a prevalent risk factor for cardiovascular disease, affects more than 1.2 billion people worldwide. It has emerged as a grave and costly public health dilemma, garnering significant attention [1]. Meta-analyses have consistently revealed a substantial association between hypertension and an elevated risk of neurological diseases such as Parkinson’s disease and stroke [2–4]. In addition, hypertension stands as a paramount predictor of mortality, exerting its influence as a global risk factor for death, disability, and years of life lost [5]. Furthermore, hypertension imposes a substantial economic burden. Despite improvements in hypertension awareness and treatment, the control rate among the hypertensive population remains low, plunging below 20–30% in several Western countries [6, 7]. Given the increasingly younger age of hypertension onset, it becomes imperative to prioritize managing the overall risk profile of patients afflicted with underlying hypertension rather than solely focusing on blood pressure (BP) measurement. Therefore, it becomes crucial to delve into the potential risk factors of hypertension and establish efficacious prevention and risk management strategies.

When examining the factors contributing to hypertension, dietary habits (specifically, high sodium intake) and unhealthy lifestyles have consistently garnered attention. More recently, studies have uncovered a significant connection between sleep disorders and various chronic conditions, including cardiovascular disease [8], chronic kidney disease [9], and cognitive impairment [10]. Both insufficient and excessive sleep duration, as well as prolonged sleep onset latency, have been associated with an elevated risk of chronic ailments [11]. Sleep patterns influence blood pressure through alterations in autonomic nervous system function and other physiological mechanisms. Unfavorable sleep conditions, such as sleep apnea, insomnia, and abnormal sleep duration, heighten the susceptibility to hypertension [12]. Nevertheless, further research is required to investigate the relationship between sleep-related issues and blood pressure across different age and gender groups.

Depression, a mood disorder classified within the psychiatric domain, imposes a significant global disease burden [13]. Notably, studies have identified cardiovascular disease as the leading cause of mortality among individuals with mental illness [14, 15]. This may be attributed to the pronounced BP fluctuations in individuals with psychiatric disorders, leading to increased cardiovascular risk [16]. Research has demonstrated a higher incidence of hypertension among patients with depression [17–19]. Furthermore, a systematic review has revealed a bidirectional relationship between sleep disorders and depression [20]. The interplay between depression and sleep disturbance augments the risk of stroke, and the risk of high blood pressure in people aged 60 years and above [21, 22]. Consequently, there may exist shared pathways between depression and sleep disturbances that exert a mutual influence on cardiovascular disease, thereby substantially increasing the risk of cardiovascular disease in people affected by both conditions. However, prior investigations have primarily focused on individuals aged 60 years and above, with limited studies encompassing diverse adult age groups. In the context of mounting societal and economic pressures, the prevalence of sleep-related disorders and depression is escalating higher among younger. Therefore, it remains imperative for clinicians to elucidate the intricate relationship among sleep disorders, depression, and hypertension. Such insights will serve to enhance the management of hypertension by effectively addressing its underlying risk factors [23, 24]. In this study, we utilized data from the National Health and Nutrition Examination Survey (NHANES), conducted by the National Center for Health Statistics (NCHS) in the United States, to explore the interaction between sleep disorders, depression, and hypertension risk. We also examined the independent and bidirectional associations between sleep disorder and depression. Furthermore, we performed a stratified analysis based on demographic factors such as age, sex, and body mass index to gain insight into potential variations within these associations.

## Methods

### Study design and population

The NHANES assesses the health and nutritional status of both adults and children in the United States. This research project employs questionnaires and physical examinations to target various population groups and health issues. Its findings help determine disease prevalence and risk factors, assess nutritional status, and understand the relationship between nutrition and health outcomes for disease prevention and health promotion. We conducted an analysis using data from the NHANES, an integral project of the National Center for Health Statistics under the Centers for Disease Control and Prevention [25]. The data was collected through a meticulous multistage probabilistic design that encompassed geographically stratified areas and proportional representation of minority populations. The NHANES database is typically managed by expert investigators affiliated with the NCHS or associated organizations. Trained extensively, these investigators oversee various survey phases, including demographic and dietary data, questionnaire survey data, laboratory examination data, and health check data. Their expertise guarantees the precision and dependability of NHANES surveys. For our analysis, we integrated seven consecutive NHANES survey cycles spanning from 2005 to 2018. Ethical approval was obtained from the National Center for Health Statistics Ethical Review Board, and all participants provided written informed consent [26].

**Fig. 1:**
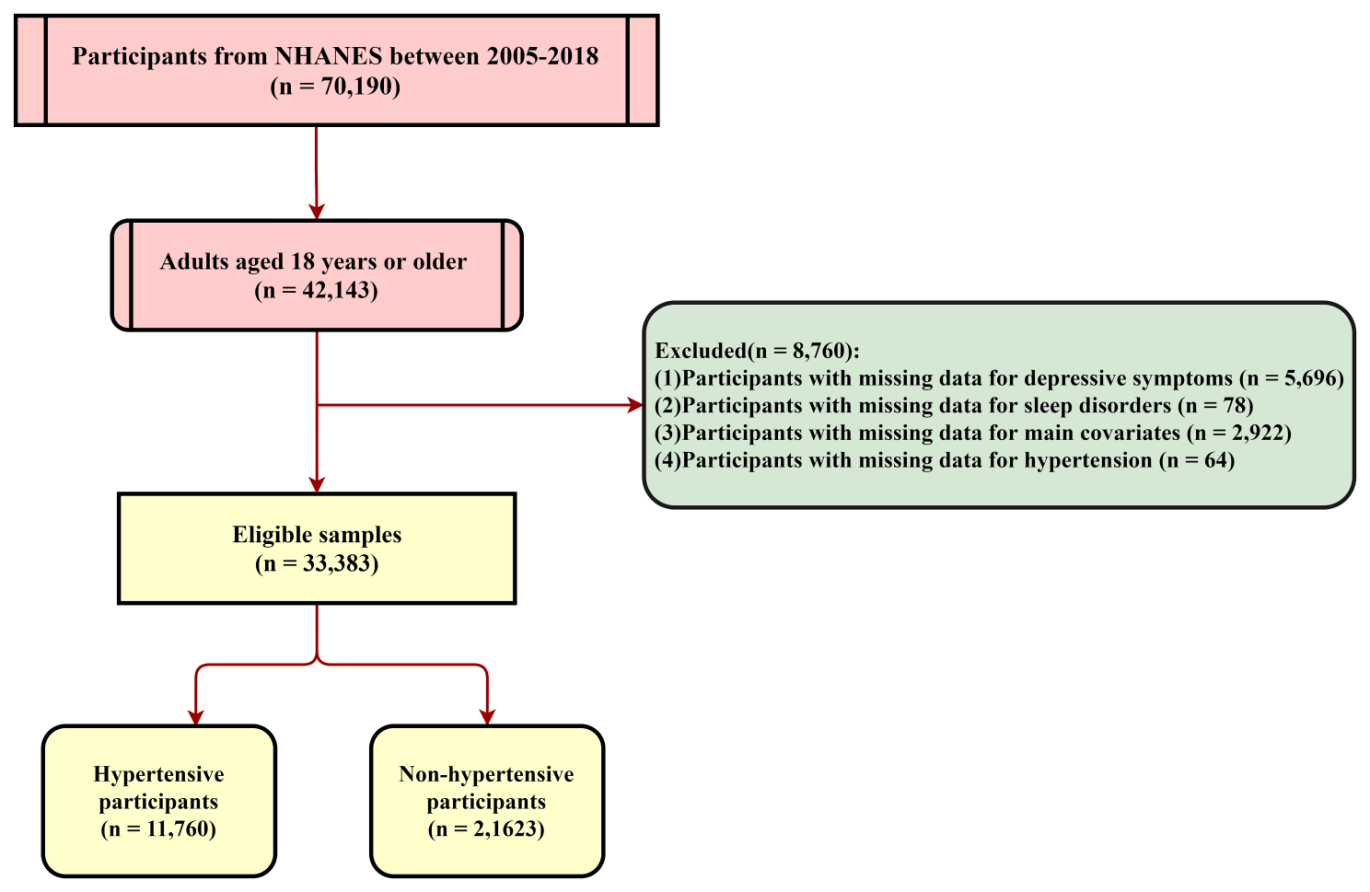
The screening process of participants in this study

This study implemented a cross-sectional design to retrieve data from the NHANES database, specifically targeting 42,143 participants aged 18 years and above across the seven survey cycles between 2005 and 2018. Exclusion criteria were applied by the study design, which included the following: [1] age < 18 years; [2] missing depression questionnaire data; [3] missing hypertension data; [4] duplicate respondents; [5] missing sleep disorder data; [5] respondents with incomplete main covariates. The selection process, as visually shown in Fig. 1, resulted in the inclusion of 33,383 participants for our study.

### Assessment of hypertension

The primary outcome variable in this study was the presence of hypertension among the participants. Hypertension was defined as previously described [27, 28]: [1] a mean systolic blood pressure ≥ 130 mmHg or a diastolic blood pressure ≥ 80 mmHg; [2] self-reported diagnosis of hypertension; [3] self-reported use of antihypertensive medication. Any of the above three conditions was indicative of a diagnosis of hypertension [29]. However, due to substantial data gaps in the other two methods, we chose questionnaire surveys as the hypertension diagnostic method. Hence, self-reporting was employed in this study to assess hypertension. Participants were asked a specific question, to which they responded affirmatively: “*Have you ever received a diagnosis of high blood pressure from a physician or other healthcare professional?*” While this question does not serve as a conclusive diagnosis of hypertension, it has been employed in epidemiological studies and has demonstrated utility in screening for hypertension [30].

### Assessment of sleep-related disorders

We evaluated several sleep-related issues, including sleep duration, sleep onset latency, sleep disorders, and sleep difficulties. These outcomes were defined based on the NHANES sleep questionnaire [31–33]: sleep duration was categorized as relatively insufficient (< 7 h/night), normal (7–8 h/night), or relatively excessive (> 8 h/night). Sleep onset latency was classified into short (< 5 min/night), normal (5–30 min/night), or long (> 30 min/night). Regarding sleep disorders, participants were identified as experiencing a sleep disorder or having difficulty sleeping if they responded affirmatively to the question, “*Have you ever reported to a physician or other healthcare professional that you encounter challenges with sleep or have a diagnosed sleep disorder?*“.

### Assessment of depression

In the NHANES database, the assessment of depressive symptoms was conducted employing the PHQ-9 screening tool, which is encompassed in the diagnostic criteria for depression outlined in the Diagnostic and Statistical Manual of Mental Disorders (DSM-IV). This screening tool has established its reliability and efficacy for both clinical and research purposes [34]. The questionnaire consists of nine questions, with each item rated on a scale from 0 to 3, yielding a total score ranging from 0 to 27. Rating options include 0 (no symptoms present), 1 (symptoms occurring over a few days), 2 (symptoms present more than half the time), and 3 (symptoms present almost daily). These questions were administered by trained interviewers at the Mobile Examination Center (MEC), a mobile medical facility utilized in the NHANES study for conducting on-site examinations and data collection. The PHQ-9 primarily captures the frequency of self-reported depressive symptoms experienced within the preceding two-week period [35]. Following DSM-IV, a PHQ-9 score of ≥ 10 was deemed indicative of a depressive symptom [36]. The severity of depression was further categorized as follows: absence of depression (PHQ-9 score: 0–9), moderate depression (PHQ-9 score: 10–14), moderate to severe depression (PHQ-9 score: 15–19), and severe depression (PHQ-9 score: 20–27) [37].

### Data collection

Demographic information was collected through questionnaires, encompassing variables such as age (categorized into 18–44 years, 45–64 years, and 65 years and older), sex, race (including non-Hispanic white, non-Hispanic black, Hispanic, etc.), educational attainment (college or lower), and the ratio of family income to poverty (classified as below 1.33, 1.33–3.50, and above 3.5), representing the proportion of household income relative to the federal poverty line, adjusted for family size. Additional covariates comprised smoking status (defined as non-smoker [less than 100 cigarettes in a lifetime], former smoker [more than 100 cigarettes in one’s lifetime, now not smoking at all], current smoker [more than 100 cigarettes in a lifetime, now smoking sometimes or daily]), and drinking status (categorized as non-drinker, 1–5 drinks/month, 5–10 drinks/month, and 10 + drinks/month) [38], body mass index (low weight [< 18.5 kg/m^2^], normal weight [18.5–25 kg/m^2^], overweight [25–30 kg/m^2^], obesity [≥ 30 kg/m^2^]), and diabetes data. The diagnostic criteria for diabetes in this study included: [1] physician-diagnosed diabetes; [2] glycosylated hemoglobin (HbA1c) level exceeding 6.5%; [3] fasting blood glucose (FBG) level of ≥ 7.0 mmol/l. Blood samples for HbA1c measurement and FBG analysis were collected by trained medical personnel according to standardized procedures during the NHANES survey. Subsequent laboratory analysis of blood samples was conducted by qualified technicians to determine the HbA1c and FBG levels. The presence of any of these three conditions signified a diabetes diagnosis.

Key study variables encompassed hypertension, sleep disorder, depression, and sleep-related problems (sleep duration: 7–8 h/night, < 7 h/night, > 8 h/night; sleep onset time: <5 min, 5–30 min, > 30 min). All participants were divided into two groups: those with hypertension (*n* = 11,760) and those without hypertension (*n* = 21,623).

### Statistical analysis

Statistical analysis in this study incorporated the complex sampling design of the NHANES database by applying weighted analysis using interview weights (WTMEC2YR) and sampling weights for study design variables (SDMVPSU and SDMVSTRA). Continuous variables were expressed as mean ± standard deviation (SD) and compared using Student’s t-test between groups. Categorical data were presented as counts and percentages [n (%)] and analyzed using the Rao-Scott chi-square test. Statistical software packages utilized for analysis included SPSS (version 23.0) and R (version 4.1.3). Multivariate logistic regression analysis was used to examine the relationship between sleep disorder, depression, and their interaction with hypertension. Model 1 was unadjusted (crude). Model 2 adjusted for sex, age, race, education level, and the ratio of family income to poverty. Model 3 added further adjustments for BMI, drinking status, smoking status, and diabetes [39, 40]. In this study, all analyses were conducted using a two-sided approach, with statistical significance set at P < 0.05. We utilized several commonly used statistical packages, including ‘stats,’ ‘gtsummary,’ ‘glm,’ and ‘survey.’ Additionally, we employed the ‘interactionR’ tool to explore relevant indicators of additive interactions. It’s worth noting that we customized these R software packages to meet the specific needs of our study, allowing us to compute additional metrics and results.

Initially, we investigate whether there is a multiplicative interaction between sleep disorders and depression concerning hypertension risk by examining their product. This evaluation aims to determine the nature of this interaction, whether it is positive or negative. To further quantify their interaction in terms of hypertension risk, we utilize the relative excess risk due to interaction (RERI), the attributable proportion (AP), and the synergy index (SI). It’s noteworthy that when the 95% confidence interval (CI) for RERI or AP excludes 0, or the 95% CI for SI excludes 1, larger absolute values of these statistics indicate a higher degree of interaction [41]. RERI quantifies the excess risk attributed to the interaction between sleep disorders and depression. AP indicates the proportion of the combined risk attributable to this interaction. SI represents the increase in risk resulting from the combined impact of both factors. An SI value greater than 1 indicates a significant synergistic effect, where the combined impact exceeds the sum of individual effects. Conversely, an SI equal to or less than 1 suggests that the joint effect does not amplify the risk as much as the individual effects combined.

## Results

### The characteristics of all participants

In this study, we utilized data from seven NHANES dataset periods, namely 2005–2006, 2007–2008, 2009–2010, 2011–2012, 2013–2014, 2015–2016, and 2017–2018. According to the study’s inclusion and exclusion criteria, a total of 33,383 participants aged 18 years and above were finally included in this analysis. The surveyed population encompassed individuals between the ages of 18 and 85 years. Among them, 14,550 (68%) participants were non-Hispanic whites, and 17,338 (52%) participants were women. Baseline characteristics were compared based on the presence or absence of hypertension, and the specific results are shown in Table 1. The average age of hypertensive patients was 57 ± 15 years, with 6,216 (53%) being males and 5,345 (70%) being non-Hispanic whites. Additionally, individuals with hypertension displayed variations in depression, sleep duration, sleep onset latency, and symptoms of sleep disorder or difficulty compared to those without hypertension. Age, sex, race, education level, ratio of family income to poverty, smoking status, drinking status, diabetes, BMI, and waist circumference also exhibited significant differences between the hypertension and non-hypertension groups (all *P* < 0.05).

**Table 1:** General characteristics of included participants according to the presence or absence of hypertension in the NHANES 2005–2018

			Group		
Characteristic	N^1^	Overall *N* = 33,383 (100%)^2^	Hypertension *N* = 11,760 (32%)^2^	Non-hypertension *N* = 21,623 (68%)^2^	P Value^3^
Age (years), Median (Mean, SD)	33,383	46 [17, 42]	58 [15, 43]	40 [16, 44]	< 0.001
Age group, n (%)	33,383				< 0.001
18–44 years		15,109 (45%)	2,074 (18%)	13,035 (60%)	
45–64 years		10,563 (32%)	4,705 (40%)	5,858 (27%)	
>= 65 years		7,711 (23%)	4,981 (42%)	2,730 (13%)	
Gender, n (%)	33,383				0.026
Female		17,338 (52%)	6,216 (53%)	11,122 (51%)	
Male		16,045 (48%)	5,544 (47%)	10,501 (49%)	
Race, n (%)	33,383				< 0.001
Mexican American		5,297 (16%)	1,347 (11%)	3,950 (18%)	
Other Hispanic		3,034 (9%)	954 (8%)	2,080 (10%)	
Non-Hispanic White		14,550 (44%)	5,345 (45%)	9,205 (43%)	
Non-Hispanic Black		7,342 (22%)	3,241 (28%)	4,101 (19%)	
Other Races		3,160 (9%)	873 (8%)	2,287 (10%)	
Education level, n (%)	31,471				< 0.001
<=High school		8,201 (26%)	3,379 (29%)	4,822 (24%)	
> High school		23,226 (74%)	8,263 (71%)	14,963 (76%)	
Missing data		44 (< 0.1%)	20 (< 0.1%)	24 (< 0.1%)	
Ratio of family income to poverty, Median (Mean, SD)	30,459	2.92 (2.96, 1.65)	2.74 (2.91, 1.62)	3.01 (2.99, 1.67)	0.031
Ratio of family income to poverty, n (%)	30,459				< 0.001
< 1.33		10,379 (34%)	3,644 (34%)	6,735 (34%)	
1.33–3.5		11,047 (36%)	4,048 (38%)	6,999 (35%)	
> 3.5		9,033 (30%)	2,985 (28%)	6,048 (31%)	
Body Mass Index, Median (Mean, SD)	31,722	28 [7, 29]	30 [7, 31]	27 [6, 28]	< 0.001
Body Mass Index group1, n (%)	31,722				< 0.001
Underweight (< 18.5)		601 (2%)	99 (< 0.1%)	502 (2%)	
Normal (18.5 to < 25)		9,087 (29%)	1,923 (17%)	7,164 (35%)	
Overweight (25 to < 30)		10,240 (32%)	3,486 (32%)	6,754 (33%)	
Obese (30 or greater)		11,794 (37%)	5,590 (50%)	6,204 (30%)	
Body Mass Index group2, n (%)	31,722				< 0.001
BMI < 30		19,928 (63%)	5,508 (50%)	14,420 (70%)	
BMI > = 30		11,794 (37%)	5,590 (50%)	6,204 (30%)	
Waist circumference, Median (Mean, SD)	30,312	97 (99, 17)	105 (106, 16)	94 (95, 16)	< 0.001
Smoking status, n (%)	33,383				< 0.001
Current		6,787 (20%)	2,230 (19%)	4,557 (21%)	
Former		7,754 (23%)	3,677 (31%)	4,077 (19%)	
Never		18,842 (57%)	5,853 (50%)	12,989 (60%)	
Alcohol consumption status, n (%)	26,669				< 0.001
Non-drinker		7,744 (29%)	3,100 (32%)	4,644 (27%)	
1–5 drinks/month		13,100 (49%)	4,546 (47%)	8,554 (50%)	
5–10 drinks/month		2,080 (8%)	564 (6%)	1,516 (9%)	
10 + drinks/month		3,738 (14%)	1,373 (14%)	2,365 (14%)	
Missing data		7 (< 0.1%)	3 (< 0.1%)	4 (< 0.1%)	
Diabetes, n (%)	33,383	5,370 (16%)	3,615 (31%)	1,755 (8%)	< 0.001
Sleep onset latency time, Median (Mean, SD)	11,755	15 [20, 22]	15 [21, 24]	15 [19, 21]	< 0.001
Sleep onset latency time, n (%)	11,755				< 0.001
< 5 min		1,215 (10%)	348 (9%)	867 (11%)	
5–30 min		8,144 (69%)	2,501 (67%)	5,643 (70%)	
> 30 min		2,396 (21%)	886 (24%)	1,510 (19%)	
Sleep duration, n (%)	30,205				< 0.001
<7 h/night		11,584 (38%)	4,270 (42%)	7,314 (36%)	
7–8 h/night		16,092 (53%)	4,892 (48%)	11,200 (56%)	
>8 h/night		2,529 (9%)	946 (9%)	1,583 (8%)	
Depression, n (%)	33,383				< 0.001
No depression		30,508 (91%)	10,384 (88%)	20,124 (93%)	
Moderate depression		2,562 (8%)	1,199 (10%)	1,363 (6%)	
Severe depression		313 (1%)	177 (2%)	136 (1%)	
Sleep disorders, n (%)	33,383				< 0.001
No sleep disorders		22,752 (68%)	6,504 (54%)	16,248 (75%)	
Sleep disorders		10,631 (32%)	5,256 (46%)	5,375 (25%)	

1 N not Missing (unweighted)

2 Median (IQR) for continuous; n (%) for categorical

3 Wilcoxon rank-sum test for complex survey samples; chi-squared test with Rao & Scott’s second-order correction

Among the participants, 11,584 (36%) reported a nightly sleep duration of less than seven hours, whereas only 2,529 (7.5%) participants reported a sleep duration exceeding eight hours. Moreover, a total of 1,215 (11%) participants indicated a rapid sleep onset, falling asleep within just five minutes or less. In contrast, 2,396 (18%) participants reported a prolonged sleep onset period, requiring more than 30 min to drift into slumber. Furthermore, 10,631 (35%) individuals reported experiencing sleep disorders or encountering difficulties in their sleep patterns. Additionally, a total of 2,875 (8%) participants reported depression, among whom 2,562 had moderate to severe depression, while 313 had severe depression.

### Comparisons of the characteristics between patients with and without hypertension

The median age of hypertensive patients is 58 years, significantly higher than that of non-hypertensive individuals (*P* < 0.001). Additionally, in the hypertension group, the proportion of individuals aged 65 and above is significantly higher compared to the non-hypertension group (42% vs. 13%, *P* < 0.001). Similarly, patients with hypertension had a median BMI of 30 kg/m², which was higher than the median BMI of 27 kg/m² observed in non-hypertensive patients (*P* < 0.001). Additionally, there are notable disparities in the distribution of BMI categories between the hypertensive and non-hypertensive groups. Specifically, in the hypertensive group, the proportion of individuals with a BMI of 30 or higher is significantly greater compared to the non-hypertensive group (50% vs. 30%, *P* < 0.001). Among hypertensive patients, the proportion of women was 53%, slightly higher than 51% of non-hypertensive patients (*P* = 0.03). The family poverty index of hypertensive patients was significantly lower than that of non-hypertensive patients (median 2.71 vs. 3.01, *P* = 0.03). Moreover, in the hypertension group, the proportion of individuals with a household income-to-poverty ratio exceeding 3.5 is lower compared to the non-hypertension group (28% vs. 31%, *P* < 0.001). The prevalence of smoking history among hypertensive patients reached 50%, significantly surpassing the 40% found in non-hypertensive patients (*P* < 0.001). Moreover, the proportion of hypertensive patients in individuals with diabetes was higher compared to non-hypertensive patients (31% vs. 8%, *P* < 0.001). Meanwhile, the prevalence of sleep disorders among hypertensive patients was 46%, significantly higher than the 25% reported among non-hypertensive patients (*P* < 0.001). Furthermore, the proportion of individuals diagnosed with hypertension among patients with depression was 12%, significantly exceeding the 7% observed in patients without hypertension (*P* < 0.001). However, the prevalence of alcohol consumption among individuals with hypertension was lower than that in the non-hypertensive population (68% vs. 73%, *P* < 0.001). Furthermore, in the hypertension group, the proportion of individuals with a higher education level is lower compared to the non-hypertension group (71% vs. 76%, *P* < 0.001) (Table 1).

### Associations of sleep disorders or depression with hypertension

Compared to individuals without sleep disorders, those with sleep disorders had a higher risk of hypertension in Model 1, after adjusting for age and sex (OR = 1.94, 95% CI: 1.77–2.11). This risk increased in Model 2 with additional adjustments for race, education level, and family income-to-poverty ratio (OR = 1.96, 95% CI: 1.79–2.15). In Model 3, which included comprehensive adjustments for various factors such as age, sex, race, education level, family income-to-poverty ratio, BMI, alcohol consumption, smoking history, and diabetes, patients with sleep disorders still showed an elevated risk of hypertension (OR = 1.62, 95% CI: 1.48–1.77).

The study also examined the relationship between sleep duration and sleep onset latency with hypertension. In Model 3, individuals sleeping less than 7 h per night had a higher risk of hypertension compared to those with 7–8 h of sleep (OR = 1.21, 95% CI: 1.11–1.32), after adjusting for various factors. Additionally, participants with a sleep onset latency of 5 to 30 min and those with a latency of more than 30 min had significantly increased risks of hypertension (OR = 1.47, 95% CI: 1.14–1.91) and (OR = 1.72, 95% CI: 1.33–2.24), respectively, compared to those with a latency of less than 5 min, after adjusting for the same variables. In summary, Table 2 provides robust statistical evidence supporting a strong association between sleep disorders, sleep duration, and sleep onset latency with the risk of hypertension.

**Table 2:** Association of sleep-related disorders and depression with hypertension

Variables	Model 1 OR (95% CI)	*p*-value	Model 2 OR (95% CI)	*p*-value	Model 3 OR (95% CI)	*p*-value
Sleep duration						
<7 h/night	1.4(1.30–1.52)	< 0.001	1.33(1.22–1.44)	< 0.001	1.21(1.11–1.32)	< 0.001
7–8 h/night	Ref		Ref		Ref	
>8 h/night	1.14(1.00–1.30)	0.045	1.1(0.96–1.27)	0.2	1.09(0.95–1.26)	0.2
Sleep-onset latency time						
< 5 min	Ref		Ref		Ref	
5–30 min	1.28(1.09–1.51)	0.004	1.32(1.10–1.59)	0.005	1.47(1.14–1.91)	0.006
> 30 min	1.75(1.47–2.09)	< 0.001	1.68(1.38–2.04)	< 0.001	1.72(1.33–2.24)	< 0.001
Sleep disorder/trouble						
No sleep disorders						
Sleep disorders	1.94(1.77–2.11)	< 0.001	1.96(1.79–2.15)	< 0.001	1.62(1.48–1.77)	< 0.001
Depression						
No depression	Ref		Ref		Ref	
Moderate depression	2.07(1.83–2.34)	< 0.001	1.94(1.69–2.22)	< 0.001	1.57(1.35–1.82)	< 0.001
Severe depression	2.82(2.01–3.95)	< 0.001	2.38(1.67–3.39)	< 0.001	1.99(1.34–2.96)	< 0.001

OR = Odds Ratio; CI = Confidence Interval; Model 1, adjustment for age and gender; Model 2, adjustment for age, gender, race, education, and ratio of family income to poverty; Model 3, adjustment for age, gender, race, education, the ratio of family income to poverty, BMI, alcohol consumption status, smoking status, and diabetes

Our investigation focused on the impact of depression on hypertension through the utilization of multiple models. In Model 1, wherein adjustments were made for age and sex, depression was associated with a higher risk of hypertension (OR = 2.14, 95% CI: 1.89–2.41). This increased risk of hypertension persisted in Model 2 (OR = 1.98, 95% CI: 1.74–2.26) and Model 3 (OR = 1.61, 95% CI: 1.39–1.87). Furthermore, our study delved into the connection between depression severity and hypertension within the confines of Model 3. Following adjustments for various factors, individuals experiencing moderate depression showcased an augmented risk of hypertension (OR = 1.57, 95% CI: 1.35–1.82), while those grappling with severe depression exhibited a significantly higher risk (OR = 1.99, 95% CI: 1.34–2.96). These findings conclusively indicated a positive correlation between depression and hypertension, with increasing depression severity associated with a higher risk of developing hypertension (Table 2).

### Interaction effects between sleep disorders and depression on hypertension

Initially, we assessed the multiple interactions between sleep disorders and depression on hypertension risk (Table 3). After incorporating sleep disorders, depression, and their product into a multivariate logistic regression model, we observed a statistically significant contribution of the product of sleep disorders and depression to hypertension risk (OR = 1.07, 95% CI: 1.02–1.13, *P* < 0.05), indicating a potential positive synergistic effect between sleep disorders and depression.

**Table 3:** Multiplicative interaction between sleep disorders and depression with hypertension

Variables	Model 1 OR (95% CI)	*p*-value	Model 2 OR (95% CI)	*p*-value	Model 3 OR (95% CI)	*p*-value
Sleep disorders	1.11(1.09–1.13)	< 0.001	1.11(1.10–1.13)	< 0.001	1.07(1.05–1.09)	< 0.001
Depression	1.06(1.03–1.10)	< 0.001	1.04(1.01–1.08)	0.048	1.01(0.97–1.05)	0.6
Depression * Sleep disorders	1.06(1.02–1.11)	0.007	1.07(1.02–1.12)	0.003	1.07(1.02–1.13)	0.005

OR = Odds Ratio; CI = Confidence Interval; Model 1, adjustment for age and gender; Model 2, adjustment for age, gender, race, education, and ratio of family income to poverty; Model 3, adjustment for age, gender, race, education, the ratio of family income to poverty, BMI, alcohol consumption status, smoking status, and diabetes

To further quantify the degree of interaction related to hypertension risk, we employed measures of RERI, AP, and SI to assess the extent of this interaction. We categorized participants into four groups based on their sleep disorders and depression status: no depression, but presence of sleep disorders; depression without sleep disorders; depression with coexisting sleep disorders; neither depression nor sleep disorders. Patients with both depression and sleep disorders had an increased risk of hypertension, with an OR of 3.30 (95% CI: 2.82–3.88) in Model 1, which remained significant even after adjusting for race, education level, and ratio of family income to poverty in Model 2 (OR = 3.11, 95% CI: 2.63–3.76) and in Model 3 (OR = 2.34, 95% CI: 1.95–2.80) (Table 4). The results in Table 4 indicate a significant synergy between sleep disorders and depression in relation to hypertension in Model 3 (RERI = 0.73, 95% CI = 0.56–0.92; AP = 0.31, 95% CI = 0.11–0.46; SI = 2.19, 95% CI = 1.08–3.46). In this context, the AP value of 0.31 in Model 3 suggests that 31% of hypertension cases in the study sample can be attributed to the interaction between sleep disorders and depression. The SI value of 2.19 indicates a significant increase in hypertension risk due to the combined effect of these two factors. The RERI value of 0.73 reveals an additional risk of 0.73 associated with the interaction between sleep disorders and depression in causing hypertension.

**Table 4:** Analysis of additive interaction between sleep disorders and depression with hypertension

Sleep disorders	Depression	Model 1 OR (95% CI)	*p*-value	Model 2 OR (95% CI)	*p*-value	Model 3 OR (95% CI)	*p*-value
No	No	Ref		Ref		Ref	
Yes	No	1.78(1.62–1.95)	< 0.001	1.82(1.65–2.00)	< 0.001	1.51(1.37–1.67)	< 0.001
No	Yes	1.45(1.19–1.76)	< 0.001	1.28(1.04–1.58)	0.021	1.10(0.86–1.40)	0.4
Yes	Yes	3.30(2.82–3.88)	< 0.001	3.11(2.63–3.67)	< 0.001	2.34(1.95–2.80)	< 0.001
RERI (95%CI)		1.07(0.92–1.26)		1.01(0.86–1.21)		0.73(0.56–0.92)	
API (95%CI)		0.32(0.13–0.47)		0.32(0.12–0.47)		0.31(0.11–0.46)	
SI (95%CI)		1.87(0.78–2.82)		1.92(0.83–2.88)		2.19(1.08–3.46)	

OR = Odds Ratio; CI = Confidence Interval; Model 1, adjustment for age and gender; Model 2, adjustment for age, gender, race, education, and ratio of family income to poverty; Model 3, adjustment for age, gender, race, education, the ratio of family income to poverty, BMI, alcohol consumption status, smoking status, and diabetes. RERI = relative excess risk of interaction; API = attribution proportion of interaction; SI = synergy index

### Subgroup analyses and sensitivity analyses

Subgroup analyses by age, BMI, and sex consistently demonstrated statistically significant risk ratios for individuals with both sleep disorders and depression (*P* < 0.05) (Tables 5 and 6). In the subgroup analysis presented in Table 5, a multiplicative interaction effect between sleep disorders and depression on hypertension was observed, aligning with the overall analysis findings. Likewise, additional subgroup analysis for additive interaction (Table 6) affirmed this observation, signaling a notable increase in hypertension risk among those concurrently affected by sleep disorders and depression. These subgroup analysis results corroborate the overarching analysis outcomes, further fortifying the credibility of the study’s findings. The findings suggest a significant interaction among sleep disorders, depression, and hypertension.

**Table 5:** Analysis of multiplicative interaction between sleep disorders and depression with hypertension in different demographic subgroups

Subgroup	Depression * Sleep disorders	OR (95% CI) Model 1	p-value	OR (95% CI) Model 2	p-value	OR (95% CI) Model 3	p-value
Age subgroup	18–44 years	1.07(1.01–1.14)	0.031	1.05(1.01–1.13)	0.033	1.06(1.01–1.13)	0.041
	45–64 years	1.02(1.00-1.16)	0.032	1.04(1.00-1.11)	0.041	1.05(1.00-1.09)	0.046
	>= 65 years	1.14(1.01–1.28)	0.03	1.18(1.04–1.34)	0.013	1.15(1.01–1.32)	0.036
Gender group	Female	1.06(1.00-1.12)	0.037	1.06(1.01–1.13)	0.033	1.06(1.00-1.12)	0.039
	Male	1.08(1.00-1.16)	0.054	1.09(1.00-1.18)	0.048	1.09(1.00-1.18)	0.039
BMI Group	BMI < 30	1.07(1.00-1.14)	0.047	1.07(1.01–1.15)	0.034	1.08(1.01–1.16)	0.029
	BMI > = 30	1.05(1.01–1.12)	0.015	1.05(1.01–1.13)	0.021	1.07(1.00-1.14)	0.046

OR = Odds Ratio; CI = Confidence Interval; Model 1, non-adjustment; Model 2, adjustment for race, education, and the ratio of family income to poverty; Model 3, adjustment for race, education, the ratio of family income to poverty, alcohol consumption status, smoking status, and diabetes

**Table 6:** Analysis of additive interaction between sleep disorders and depression with hypertension in different demographic subgroups

Subgroup		Sleep disorders	Depression	OR (95% CI) Model 1	*p*-value	OR (95% CI) Model 2	*p*-value	OR (95% CI) Model 3	*p*-value
Age subgroup	18–44 years	No	No	—		—		—	
		Yes	No	1.12(1.10–1.14)	< 0.001	1.12(1.10–1.14)	< 0.001	1.08(1.05–1.11)	< 0.001
		No	Yes	1.04(1.00–1.08)	0.041	1.04(1.0–1.08)	0.084	1.01(0.97–1.06)	0.5
		Yes	Yes	1.25(1.19–1.32)	< 0.001	1.23(1.16–1.29)	< 0.001	1.16(1.10–1.22)	< 0.001
	45–64 years	No	No	—		—		—	
		Yes	No	1.15(1.11–1.19)	< 0.001	1.15(1.11–1.20)	< 0.001	1.10(1.06–1.14)	< 0.001
		No	Yes	1.12(1.05–1.19)	< 0.001	1.07(1.01–1.15)	0.034	1.04(0.97–1.12)	0.3
		Yes	Yes	1.31(1.24–1.39)	< 0.001	1.28(1.21–1.37)	< 0.001	1.19(1.12–1.27)	< 0.001
	>= 65 years								
		No	No	—		—		—	
		Yes	No	1.06(1.03–1.10)	< 0.001	1.07(1.04–1.11)	< 0.001	1.05(1.01–1.09)	0.014
		No	Yes	0.99(0.90–1.08)	0.8	0.95(0.86–1.05)	0.3	0.93(0.84–1.03)	0.2
		Yes	Yes	1.2(1.12–1.28)	< 0.001	1.2(1.12–1.29)	< 0.001	1.13(1.05–1.21)	0.002
Gender subgroup	Female								
		No	No	—		—		—	
		Yes	No	1.09(1.07–1.11)	< 0.001	1.10(1.07–1.12)	< 0.001	1.07(1.05–1.09)	< 0.001
		No	Yes	1.09(1.04–1.14)	< 0.001	1.06(1.01–1.11)	0.026	1.02(0.97–1.07)	0.4
		Yes	Yes	1.26(1.21–1.30)	< 0.001	1.23(1.19–1.28)	< 0.001	1.16(1.12–1.20)	< 0.001
	Male								
		No	No	—		—		—	
		Yes	No	1.13(1.10–1.16)	< 0.001	1.13(1.10–1.16)	< 0.001	1.08(1.05–1.11)	0.2
		No	Yes	1.02(0.97–1.08)	0.4	1.00(0.95–1.07)	0.9	1.00(0.94–1.06)	> 0.9
		Yes	Yes	1.24(1.18–1.32)	< 0.001	1.24(1.16–1.31)	< 0.001	1.17(1.10–1.24)	< 0.001
BMI subgroup	BMI < 30								
		No	No	—		—		—	
		Yes	No	1.06(1.04–1.08)	< 0.001	1.07(1.05–1.08)	< 0.001	1.05(1.03–1.07)	< 0.001
		No	Yes	1.05(1.01–1.09)	0.015	1.04(1.00–1.08)	0.078	1.02(0.98–1.07)	0.3
		Yes	Yes	1.19(1.13–1.25)	< 0.001	1.13(1.13–1.25)	< 0.001	1.17(1.10–1.23)	< 0.001
	BMI > = 30								
		No	No	—		—		—	
		Yes	No	1.14(1.11–1.17)	< 0.001	1.14(1.11–1.17)	< 0.001	1.12(1.09–1.15)	< 0.001
		No	Yes	1.05(1.00–1.11)	0.061	1.04(0.99–1.10)	0.1	1.02(0.97–1.07)	0.5
		Yes	Yes	1.26(1.21–1.31)	< 0.001	1.26(1.21–1.31)	< 0.001	1.22(1.16–1.27)	< 0.001

OR = Odds Ratio; CI = Confidence Interval; Model 1, non-adjustment; Model 2, adjustment for race, education, and ratio of family income to poverty; Model 3, adjustment for race, education, the ratio of family income to poverty, alcohol consumption status, smoking status, and diabetes

Finally, we extensively examined sleep-related factors, including sleep duration and onset latency, among different demographic subgroups (Additional files 1 and 2). Among adults under 44 years, sleeping less than 7 h was significantly associated with a higher risk of hypertension compared to those who slept for 7 to 8 h (OR = 1.05, 95% CI: 1.04–1.06). Interestingly, individuals aged 65 years and older who slept for over eight hours also had a significantly increased risk of hypertension in comparison to those with 7 to 8 h of sleep (OR = 1.06, 95% CI: 1.01–1.12). Subgroup analyses consistently showed that individuals with a sleep onset latency over 30 min had a higher risk of hypertension compared to those with a latency of under 5 min (*P* < 0.05) (Additional file 1). Additionally, an investigation into the relationship between depression severity and hypertension risk across subgroups revealed a consistent trend: individuals with depression had an increased risk of hypertension, and this risk increased with the severity of depression (Additional file 2).

## Discussion

Our study, involving 33,383 participants, aimed to investigate the relationship between sleep disorders, depression, and the prevalence of hypertension. The results indicate a correlation between depression, sleep disorders, and an increased prevalence of hypertension. Moreover, we identified a potential interaction between depression and sleep disorders in the development of hypertension. Additionally, subgroup analyses based on gender, age, and BMI consistently revealed an interaction between sleep disorders and depression in the prevalence of hypertension, underscoring the robustness of our findings. These results provide a foundation for further research into the association between depression, sleep disorders, and hypertension.

The typical diagnostic criteria for hypertension are a systolic blood pressure (SBP) exceeding 130mmHg and/or a diastolic blood pressure (DBP) exceeding 80mmHg. It’s worth noting that this clinical standard may sometimes be influenced by the white coat effect, where anxiety in a medical setting can elevate blood pressure. In our study, 11,760 individuals were diagnosed with hypertension out of the total population, indicating a prevalence of approximately 35%. Our findings are in line with recent trends in hypertension prevalence among U.S. adults, which have risen significantly from 33.53 to 40.58% over the past decade [44]. These results suggest that while self-reported questionnaires were used for hypertension diagnosis in our study, the consistency between our observed hypertension prevalence and previous epidemiological survey results underscores the viability of using such questionnaires for hypertension diagnosis in our study. Additionally, around 8% of participants reported experiencing depression, aligning with the pre-COVID-19 prevalence of depression among U.S. adults as found in a study investigating depression rates and associated risk factors [45]. Furthermore, in this study, approximately 35% reported experiencing sleep disorders or difficulties with their sleep patterns, consistent with findings from an assessment of sleep habits and sleep disorders among U.S. adults from 2017 to 2020 [46]. The above results indicate that the epidemiological data on hypertension, sleep disorders, and depression in this study are consistent with previous research findings, demonstrating a high level of alignment with real-world situations. This underscores the reliability and applicability of our study results.

Globalization and rapid social and cultural changes have brought about significant alterations and new challenges, leading to substantial social and psychological pressures. In addition to inherent biological or genetic factors, factors such as job stress, financial limitations, stress from racial discrimination, depression, and anxiety can all play crucial roles in the development of cardiovascular diseases [47]. Psychological stress has been linked to an augmented susceptibility to hypertension, with research elucidating an elevated hypertension risk among individuals afflicted with depression [17, 42, 48]. Our study found a strong connection between depression and a heightened risk of hypertension, which increased alongside the severity of depression. These results support previous findings, reinforcing the notion that depression contributes to the likelihood of developing hypertension [17]. Plausible explanations for this association may be traced to the dysfunction of the hypothalamic-pituitary-adrenal (HPA) axis and heightened sympathetic activation observed in individuals manifesting depressive symptoms [49]. The HPA axis, a pivotal regulatory mechanism governing stress response and stress coping, facilitates the production and release of adrenal cortex hormones using the corticotropin-releasing factor (CRF) and adrenocorticotropic hormone (ACTH), consequently engendering the occurrence of hypertension. Moreover, multiple reviews have substantiated that the sympathetic nervous system assumes a crucial role in the pathophysiological response to stress-related hypertension [50].

Our study revealed an association between sleep disorders and an augmented vulnerability to hypertension [51]. Sleep disorders are characterized by disruptions to normal sleep patterns, including sleep onset, maintenance, or duration difficulties. Among these, difficulties with sleep onset and inadequate sleep duration are commonly observed issues and represent primary manifestations of sleep disorders. Insufficient or excessive sleep, prolonged sleep latency, sleep disorders, and difficulties may be associated with hypertension. Our findings indicate a significant rise in hypertension risk among those experiencing sleep deprivation, aligning with prior research. Additionally, individuals with a sleep onset latency exceeding 5 min show an increased risk of hypertension, consistent with a meta-analysis by Itani Osamu et al. [52]. Subgroup analysis, based on age, sex, and BMI, shows that sleeping less than 7 h raises hypertension risk among individuals aged 18–44 years. Conversely, those aged 65 and above with 8 or more hours of sleep face a heightened risk of hypertension. This underscores how both inadequate and excessive sleep increases high blood pressure risk across age groups, aligning with Guo et al.‘s meta-analysis showing a positive association between excessive sleep duration and hypertension risk [53].

While a robust correlation between sleep duration and hypertension exists, causation remains elusive in existing studies. Epidemiological data presents inconsistent associations between sleep duration and adverse health outcomes across different age and sex groups. Most studies rely on self-reported data, potentially affected by misreporting, particularly among individuals with chronic illnesses. Additionally, a prospective cohort study observed a higher incidence of hypertension among workers with a history of shift work [54, 55]. Changes in sleep patterns may disrupt the nocturnal blood pressure drop, leading to heightened sympathetic activity and hypertension. Subgroup analyses by sex revealed that sleep-deprived women had a higher hypertension risk, while men with insufficient or excessive sleep also faced increased risk. Results suggest that sleeping over 7 h may protect against hypertension in women but pose a risk for men. This study’s value lies in its ability to stratify the relationship between hypertension and sleep by sex, aligning with epidemiological findings showing a stronger association between sleep deprivation and hypertension in women [56, 57]. Subgroup analyses showed a weakening of the inverse relationship observed in the overall adult population with age. Additionally, sleep-deprived women faced an elevated risk of developing high blood pressure across their lifespan.

Depression and sleep disorders often coexist with other physical or mental health issues rather than occurring alone. Declining sleep quality can lead to elevated blood pressure, weakened immunity, and psychological problems. Depression may also contribute to sleep difficulties and anxiety [43]. Our study found a strong link between depression and hypertension. It’s been widely observed that individuals with hypertension often have concurrent sleep disorders and depression [58, 59]. Depression can incite high blood pressure, while high blood pressure can exacerbate depressive symptoms. Presently, research unequivocally underscores a connection between high blood pressure and depression [60, 61]. The link between hypertension and depression may stem from shared physiological mechanisms risk factors, or both. However, the precise mechanisms remain unclear. Research on immune system inflammation may offer insights into a common underlying mechanism, alongside potential interconnected pathways. Emotional distress, such as anxiety and depression, can exacerbate blood pressure fluctuations in hypertensive patients, creating a harmful cycle that disrupts blood pressure regulation. A study indicates that individuals with uncontrolled hypertension are significantly more susceptible to depression, highlighting the intricate interplay between these conditions [58]. This may be attributed to depressed patients’ suboptimal adherence to medication, leading to inadequate blood pressure management.

This study revealed a synergistic interaction between depression and sleep disorders, significantly influencing hypertension development. Prior research has confirmed a robust correlation between sleep disorders and various mental and psychosomatic disorders [62, 63]. Prospective cohorts have demonstrated a close association between both isolated sleep disorders and sleep disorders accompanied by depression and the risk of hypertension [64]. Positive factors like well-being, emotional stability, and life satisfaction typically enhance sleep quality, while negative factors such as poor well-being, anxiety, depression, and anger can diminish sleep quality [65]. A discontented mood can easily lead to sleep disorders, which can significantly impact both physical and mental well-being. The combined influence of depression and sleep disorders outweighs that of either condition alone. Inflammation is a key factor in depression, sleep disorders, and cardiovascular disease, all sharing common mechanisms and risk factors. Thus, physicians should prioritize enhancing patients’ sleep quality and mental health, intervening actively in psychological disorders, particularly depressive symptoms, alongside routine pharmacotherapy for hypertension.

## Limitations

This study has several limitations. Firstly, being a cross-sectional study, it only allows us to determine the association between depression or sleep disorders and hypertension, without establishing causality definitively. Secondly, the definitions of sleep disorders and hypertension relied on self-reported data from NHANES participants, possibly introducing bias into the analysis. Thirdly, subgroup analysis was limited to gender, race, and age, warranting further investigation into hypertension subgroups to understand the interaction effects of depression and sleep disorders on hypertension across diverse populations. Fourth, although we employed the method of multiple testing in data analysis, we must be cautious about its potential impact, such as the increased likelihood of discovering statistical significance, thus possibly leading to false positive results. In conclusion, our study only examined sleep disorders as a general category without distinguishing specific types like sleep apnea or insomnia, which may have varying associations with hypertension. Furthermore, the dataset lacked data on potential confounding factors such as medication usage or comorbidities, which might have impacted the observed associations. Hence, although our study offers valuable insights into the link between sleep disorders and hypertension, further longitudinal research is required to understand the underlying mechanisms and causal relationships more thoroughly.

## Conclusions

Our study investigated the impact of depression and sleep disorders on the prevalence of hypertension, utilizing data from 33,383 NHANES participants. The results unmistakably indicate that both depression and sleep disorders independently increase the prevalence of hypertension. Furthermore, our analysis reveals an interaction between depression and sleep disorders regarding hypertension prevalence, suggesting a synergistic effect. This underscores the significance of concurrent sleep disorders and depression in the development of hypertension.

### Electronic supplementary material

Below is the link to the electronic supplementary material.

Supplementary Material 1

Supplementary Material 2

Supplementary Material 3

Supplementary Material 4

Supplementary Material 5

## Data Availability

The datasets generated and/or analyzed during the current study are available in the NHANES repository, https://www.cdc.gov/nchs/nhanes/. The dataset supporting the conclusions of this article is included in Additional file 3.

## Abbreviations

NHANES: National Health and Nutritional Examination Survey
RERI: Relative excess risk due to interaction
API: Attributable proportion of interaction
SI: Synergy index
CRF: Cotropin-releasing factor
ACTH: Adrenocorticotropic hormone
BP: Blood pressure
PHQ-9: Patient Health Questionnaire
HPA: Hypothalamic-pituitary-adrenal-axis
NCHS: National Center for Health Statistics
CDC: Centers for Disease Control and Prevention
BMI: Body mass index
DSM-IV: Diagnostic and statistical manual of mental disorders
FBG: Fasting blood glucose
SD: Standard deviation
OR: Odds ratio
CI: Confidence interval
SBP: Systolic blood pressure
DBP: Diastolic blood pressure
