# Supplementary material for: Interaction effects between sleep-related disorders and depression on hypertension among adults: a cross-sectional study
Source: BMC Psychiatry. 2024 Jul 2;24:482. doi: 10.1186/s12888-024-05931-9 (PMC11221077; doi:10.1186/s12888-024-05931-9)
Supplement: Supplementary file 2 — Supplementary Material 2 [file 12888_2024_5931_MOESM2_ESM.docx]

Table S1. Subgroup analysis of sleep duration and sleep-onset latency time associations with hypertension.

|  | Sleep duration OR (95% CI) | | | | |  | Sleep-onset latency time OR (95% CI) | | | |
| --- | --- | --- | --- | --- | --- | --- | --- | --- | --- | --- |
|  |  | <7 vs. 7–8 h | p-value | >8 vs. 7–8 h | p-value |  | 5–30 min vs. <5 | p-value | > 30min vs. <5 | p-value |
| Age subgroup | 18 - 44 years |  |  |  |  |  |  |  |  |  |
|  | model 1 | 1.07(1.05 - 1.08) | <0.001 | 1(0.98 - 1.02) | 0.8 |  | 1.02(0.98 - 1.07) | 0.3 | 1.06(1.02 - 1.11) | 0.01 |
|  | model 2 | 1.06(1.05 - 1.08) | <0.001 | 1(0.98 - 1.03) | 0.8 |  | 1.03(0.98 - 1.08) | 0.3 | 1.06(1.01 - 1.12) | 0.018 |
|  | model 3 | 1.05(1.04 - 1.06) | <0.001 | 1.01(0.98 - 1.03) | 0.7 |  | 1.04(0.99 - 1.09) | 0.12 | 1.07(1.01 - 1.12) | 0.027 |
|  | 45 - 64 years |  |  |  |  |  |  |  |  |  |
|  | model 1 | 1.05(1.01 - 1.08) | 0.005 | 1.03(0.97 - 1.10) | 0.4 |  | 1.04(0.97 - 1.11) | 0.2 | 1.14(1.04 - 1.24) | 0.005 |
|  | model 2 | 1.03(0.99 - 1.06) | 0.11 | 1.01(0.95 - 1.08) | 0.8 |  | 1.05(0.98 - 1.12) | 0.14 | 1.12(1.02 - 1.22) | 0.024 |
|  | model 3 | 1(0.97 - 1.03) | >0.9 | 1(0.94 - 1.06) | >0.9 |  | 1.1(1.02 - 1.18) | 0.018 | 1.15(1.03 - 1.27) | 0.015 |
|  | >= 65 years |  |  |  |  |  |  |  |  |  |
|  | model 1 | 1.06(1.02 - 1.09) | 0.004 | 1.08(1.03 - 1.13) | 0.001 |  | 1.07(1.00 - 1.15) | 0.039 | 1.12(1.01 - 1.24) | 0.037 |
|  | model 2 | 1.04(1.01 - 1.08) | 0.023 | 1.07(1.02 - 1.12) | 0.004 |  | 1.06(1.00 - 1.13) | 0.054 | 1.09(0.98 - 1.21) | 0.12 |
|  | model 3 | 1.03(1.00 - 1.07) | 0.076 | 1.06(1.01 - 1.12) | 0.019 |  | 1.05(0.98 - 1.12) | 0.13 | 1.04(0.93 - 1.15) | 0.5 |
| Gender group | Female |  |  |  |  |  |  |  |  |  |
|  | model 1 | 1.06(1.04 - 1.08) | <0.001 | 1.02(1.00 - 1.05) | 0.1 |  | 1.04(1.00 - 1.09) | 0.039 | 1.12(1.07 - 1.18) | <0.001 |
|  | model 2 | 1.05(1.02 - 1.07) | <0.001 | 1.01(0.98 - 1.04) | 0.5 |  | 1.05(1.00 - 1.10) | 0.04 | 1.1(1.05 - 1.16) | <0.001 |
|  | model 3 | 1.02(1.00 - 1.04) | 0.021 | 1.01(0.98 - 1.04) | 0.6 |  | 1.05(0.99 - 1.12) | 0.073 | 1.09(1.02 - 1.16) | 0.014 |
|  | Male |  |  |  |  |  |  |  |  |  |
|  | model 1 | 1.05(1.03 - 1.07) | <0.001 | 1.06(1.03 - 1.10) | 0.001 |  | 1.05(1.02 - 1.09) | 0.004 | 1.08(1.03 - 1.14) | 0.003 |
|  | model 2 | 1.05(1.03 - 1.07) | <0.001 | 1.06(1.02 - 1.10) | 0.007 |  | 1.06(1.02 - 1.10) | 0.004 | 1.08(1.03 - 1.14) | 0.006 |
|  | model 3 | 1.03(1.01 - 1.05) | 0.002 | 1.05(1.01 - 1.10) | 0.019 |  | 1.08(1.04 - 1.13) | <0.001 | 1.1(1.03 - 1.16) | 0.004 |
| BMI group | BMI < 30 |  |  |  |  |  |  |  |  |  |
|  | model 1 | 1.04(1.03 - 1.06) | <0.001 | 1.05(1.03 - 1.08) | <0.001 |  | 1.05(1.02 - 1.08) | 0.004 | 1.09(1.05 - 1.14) | <0.001 |
|  | model 2 | 1.04(1.02 - 1.06) | <0.001 | 1.05(1.02 - 1.08) | <0.001 |  | 1.05(1.02 - 1.08) | 0.005 | 1.08(1.04 - 1.13) | <0.001 |
|  | model 3 | 1.04(1.02 - 1.06) | <0.001 | 1.04(1.01 - 1.08) | 0.005 |  | 1.05(1.01 - 1.09) | 0.01 | 1.07(1.02 - 1.12) | 0.009 |
|  | BMI >= 30 |  |  |  |  |  |  |  |  |  |
|  | model 1 | 1.05(1.02 - 1.07) | <0.001 | 1.01(0.96 - 1.05) | 0.8 |  | 1.07(1.01 - 1.14) | 0.018 | 1.16(1.11 - 1.21) | <0.001 |
|  | model 2 | 1.04(1.01 - 1.06) | 0.009 | 1(0.95 - 1.05) | 0.9 |  | 1.07(1.01 - 1.14) | 0.022 | 1.15(1.09 - 1.20) | <0.001 |
|  | model 3 | 1.02(1.00 - 1.05) | 0.079 | 0.99(0.94 - 1.04) | 0.7 |  | 1.09(1.02 - 1.17) | 0.016 | 1.14(1.08 - 1.20) | <0.001 |

OR = Odds Ratio; CI = Confidence Interval; Model 1, adjustment for age and gender; Model 2, adjustment for age, gender, race, education, and ratio of family income to poverty; Model 3, adjustment for age, gender, race, education, ratio of family income to poverty, BMI, alcohol consumption status, smoking status, and diabetes.
