# Supplementary material for: Interaction effects between sleep-related disorders and depression on hypertension among adults: a cross-sectional study
Source: BMC Psychiatry. 2024 Jul 2;24:482. doi: 10.1186/s12888-024-05931-9 (PMC11221077; doi:10.1186/s12888-024-05931-9)
Supplement: Supplementary file 3 — Supplementary Material 3 [file 12888_2024_5931_MOESM3_ESM.docx]

Table S2. Subgroup analysis of depression associations with hypertension.

|  |  | Depression OR (95% CI) | | |  |
| --- | --- | --- | --- | --- | --- |
|  |  | Moderate depression vs.  No depression | p-value | Severe depression vs.  No depression | p-value |
| Age  subgroup | 18 - 44 years |  |  |  |  |
|  | model 1 | 1.13(1.09 - 1.17) | <0.001 | 1.21(1.09 - 1.34) | <0.001 |
|  | model 2 | 1.12(1.07 - 1.17) | <0.001 | 1.17(1.05 - 1.30) | 0.004 |
|  | model 3 | 1.07(1.03 - 1.12) | <0.001 | 1.09(0.99 - 1.21) | 0.092 |
|  | 45 - 64 years |  |  |  |  |
|  | model 1 | 1.18(1.13 - 1.24) | <0.001 | 1.23(1.10 - 1.39) | <0.001 |
|  | model 2 | 1.09(1.02 - 1.16) | 0.009 | 1.3(1.17 - 1.44) | <0.001 |
|  | model 3 | 1.03(0.97 - 1.09) | 0.3 | 1.27(1.11 - 1.45) | <0.001 |
|  | >= 65 years |  |  |  |  |
|  | model 1 | 1.09(1.03 - 1.16) | 0.002 | 1.29(1.17 - 1.43) | <0.001 |
|  | model 2 | 1.09(1.02 - 1.16) | 0.009 | 1.3(1.17 - 1.44) | <0.001 |
|  | model 3 | 1.03(0.97 - 1.09) | 0.3 | 1.27(1.11 - 1.45) | <0.001 |
| Gender  subgroup | Female |  |  |  |  |
|  | model 1 | 1.16(1.13 - 1.20) | <0.001 | 1.22(1.12 - 1.32) | <0.001 |
|  | model 2 | 1.14(1.10 - 1.18) | <0.001 | 1.17(1.07 - 1.28) | <0.001 |
|  | model 3 | 1.08(1.05 - 1.12) | <0.001 | 1.11(1.01 - 1.22) | 0.002 |
|  | Male |  |  |  |  |
|  | model 1 | 1.12(1.07 - 1.17) | <0.001 | 1.23(1.09 - 1.39) | <0.001 |
|  | model 2 | 1.11(1.06 - 1.17) | <0.001 | 1.18(1.04 - 1.35) | 0.012 |
|  | model 3 | 1.08(1.03 - 1.13) | 0.002 | 1.13(0.99 - 1.29) | 0.06 |
| BMI  subgroup | BMI < 30 |  |  |  |  |
|  | model 1 | 1.1(1.06 - 1.15) | <0.001 | 1.22(1.10 - 1.36) | <0.001 |
|  | model 2 | 1.09(1.05 - 1.13) | <0.001 | 1.18(1.06 - 1.32) | 0.003 |
|  | model 3 | 1.07(1.03 - 1.12) | <0.001 | 1.16(1.05 - 1.30) | 0.006 |
|  | BMI >= 30 |  |  |  |  |
|  | model 1 | 1.14(1.10 - 1.18) | <0.001 | 1.17(1.07 - 1.29) | 0.001 |
|  | model 2 | 1.13(1.09 - 1.17) | <0.001 | 1.14(1.03 - 1.26) | 0.013 |
|  | model 3 | 1.08(1.04 - 1.13) | <0.001 | 1.08(0.97 - 1.21) | 0.2 |

OR = Odds Ratio; CI = Confidence Interval; Model 1, adjustment for age and gender; Model 2, adjustment for age, gender, race, education, and ratio of family income to poverty; Model 3, adjustment for age, gender, race, education, ratio of family income to poverty, BMI, alcohol consumption status, smoking status, and diabetes.
