# Supplementary material for: Interaction effects between sleep-related disorders and depression on hypertension among adults: a cross-sectional study
Source: BMC Psychiatry. 2024 Jul 2;24:482. doi: 10.1186/s12888-024-05931-9 (PMC11221077; doi:10.1186/s12888-024-05931-9)
Supplement: Supplementary file 4 — Supplementary Material 4 [file 12888_2024_5931_MOESM4_ESM.docx]

The main purpose of this R code is to compute various statistical metrics related to interaction effects in a statistical model. These metrics include the Relative Excess Risk due to Interaction (RERI), the attributable proportion (AP), the Synergy Index (SI), and the Multiplicative Effect. The code proceeds through the following steps:

1. Define a Generic Function MUM_Interaction_svyglm: This function takes a model object and the indices of the interaction variables in the model as parameters. It then computes various statistical metrics for the interaction, including RERI, AP, SI, and the multiplicative effect.

2. Extract Model Coefficients and Standard Errors: Coefficients and standard errors are extracted from the model object. These values are essential for subsequent calculations.

3. Compute RERI: By appropriately combining coefficients of the interaction term and relevant main effects, the code calculates the RERI along with its 95% confidence interval.

4. Compute AP: By appropriately combining coefficients of the interaction term and main effects, the code calculates the AP along with its 95% confidence interval.

5. Compute SI: By appropriately combining coefficients of the interaction term and main effects, the code calculates the SI along with its 95% confidence interval.

6. Compute Multiplicative Effect: The code calculates the multiplicative effect of the interaction term along with its 95% confidence interval.

7. Output Results: The computed statistical metrics are stored in data frames and returned as elements in a list.

In conclusion, the purpose of the entire code block is to perform interaction effect statistical computations in a more generic and modular way, enhancing code reusability. The final step involves using the defined function, providing a model object (`model`), and the indices of the interaction variables in the model (`coef.index`). The function is then called to obtain the statistical results for interaction effects.
